# A Randomized Controlled Trial of a Personalized Feedback Intervention for Problem Gamblers

**DOI:** 10.1371/journal.pone.0031586

**Published:** 2012-02-14

**Authors:** John A. Cunningham, David C. Hodgins, Tony Toneatto, Michelle Murphy

**Affiliations:** 1 Centre for Addiction and Mental Health, Toronto, Ontario, Canada; 2 Department of Psychology, University of Toronto, Toronto, Ontario, Canada; 3 Dalla Lana School of Public Health, University of Toronto, Toronto, Ontario, Canada; 4 Department of Psychiatry, University of Toronto, Toronto, Ontario, Canada; 5 Department of Psychology, University of Calgary, Calgary, Alberta, Canada; University of Granada, Spain

## Abstract

**Background:**

Personalized feedback is a promising self-help for problem gamblers. Such interventions have shown consistently positive results with other addictive behaviours, and our own pilot test of personalized normative feedback materials for gamblers yielded positive findings. The current randomized controlled trial evaluated the effectiveness, and the sustained efficacy, of the personalized feedback intervention materials for problem gamblers.

**Methodology/Principal Findings:**

Respondents recruited by a general population telephone screener of Ontario adults included gamblers with moderate and severe gambling problems. Those who agreed to participate were randomly assigned to receive: 1) the full personalized normative feedback intervention; 2) a partial feedback that contained all the feedback information provided to those in condition 1 but without the normative feedback content (i.e., no comparisons provided to general population gambling norms); or 3) a waiting list control condition. The primary hypothesis was that problem gamblers who received the personalized normative feedback intervention would reduce their gambling more than problem gamblers who did not receive any intervention (waiting list control condition) by the six-month follow-up.

**Conclusions/Significance:**

The study found no evidence for the impact of normative personalized feedback. However, participants who received, the partial feedback (without norms) reduced the number of days they gambled compared to participants who did not receive the intervention. We concluded that personalized feedback interventions were well received and the materials may be helpful at reducing gambling. Realistically, it can be expected that the personalized feedback intervention may have a limited, short term impact on the severity of participants' problem gambling because the intervention is just a brief screener. An Internet-based version of the personalized feedback intervention tool, however, may offer an easy to access and non-threatening portal that can be used to motivate participants to seek further help online or in person.

**Trial Registration:**

ClinicalTrials.gov NCT00578357

## Introduction

Only about one in ten gamblers with a lifetime diagnosis of gambling dependence will ever seek treatment [1]. Many of these problem gamblers are unwilling to access treatment, often because of stigma, embarrassment or a desire to handle their problems on their own [2], [3]. These problem gamblers can be helped. Research has demonstrated the effectiveness of self-help interventions for gambling problems [4], [5]. This area deserves more attention because it addresses a cost-effective means of helping problem gamblers without requiring them to come to treatment. The aim of self-help interventions is to help problem gamblers where they are, thus circumventing many of the barriers associated with traditional treatment.

To-date, research on self-help interventions for problem gamblers has focused on evaluating the efficacy of materials that guide individuals through a series of exercises to help them deal with their gambling. In effect, such interventions are providing standard treatment in a book format [6], [7]. While the book format method is effective, an important question to ask is: are there other means of providing help for problem gamblers not willing to seek formal treatment? A self-help method found effective with other addictive behaviours is personalized normative feedback summaries. Provision of summaries of their own gambling activities that compare their gambling to that of others in the general population would allow problem gamblers to evaluate their own gambling behaviour. This normative feedback technique is one of the central elements of Motivational Interviewing [8] and has been consistently found to have an impact on a variety of different substance use concerns. Personalized feedback has been found to promote behaviour change in drinkers [9], [10], [11], [12], [13], [14] and smokers [15], [16]. In drinkers, normative feedback is theorized to promote changes in alcohol use because many heavy drinkers overestimate the consumption of others. Consequently, normative feedback acts as a powerful source of social comparison motivating heavy drinkers to re-evaluate their consumption patterns [17]. The same motivational principles are hypothesized to promote change in other addictive behaviours [8]. Dr. Sanchez-Craig and colleagues [18], [19] speculated on another reason why personalized feedback interventions might be effective in reducing addictive behavior. After finding that provision of personalized feedback to problem drinkers in addition to a self-help book resulted in a greater reduction in drinking (as compared to a self-help book alone condition), it was hypothesized that one of the reasons the feedback had an impact was that it made the amount the person drank explicit. Thus, for gamblers, personalized normative feedback might also work because it makes the amount the person gambles explicit. Personalized normative feedback includes, by its nature, information that makes the amount a person gambles explicit. However, it is possible to provide personalized feedback that makes the amount a person gambles explicit without including normative comparisons. It is important to evaluate whether personalized normative feedback works because of the normative comparisons element or just because the intervention provides a summary of the recipient's gambling. Thus, the present study compared two types of personalized feedback – summaries with or without norms included – to a no intervention condition.

Gamblers often overestimate how much others are gambling [20]. The existence of this normative fallacy is the key condition that is required for personalized feedback to work. We predicted that, as for problem drinkers and smokers, when presented with normative information showing that most people gamble less than they do, gamblers would be motivated to re-evaluate their gambling behaviour(s) and reduce the amount they gamble. In fact, several authors have posited that personalized feedback interventions would work for problem gamblers [20], [21]. In addition, a pilot study conducted by our research team has provided positive evidence of the potential of this brief intervention [22]. In that pilot study, 61 respondents were recruited from an ongoing gambling research trial to help us “develop and evaluate self-help materials for gamblers.” Respondents who agreed to participate were randomly assigned to receive a personalized feedback summary or to a waiting list control. At the three-month follow-up (80.3% follow-up rate, N = 49), after controlling for baseline demographic characteristics and gambling severity, respondents in the feedback condition displayed some evidence that they were spending less money on gambling as compared to those in the control condition (*p*<.05). Participants in the intervention group were losing 75% less overall than the control group and their maximum amount gambled on average was 50% smaller. The pilot test also explored respondents' reactions to the feedback materials. Ratings of the usefulness of the feedback summary were positive and almost all recipients (96%) recommended that they be made available to other gamblers interested in evaluating or modifying their gambling.

The current randomized controlled trial evaluated the effectiveness, and the sustained efficacy, of the personalized feedback intervention materials for problem gamblers that had been used in the pilot study. The primary hypothesis was that problem gamblers who received the personalized normative feedback intervention would reduce their gambling more than problem gamblers who did not receive any intervention (waiting list control condition). In addition, we wished to explore the extent to which the normative component of the personalized feedback intervention was the active ingredient of this brief intervention. The hypothesis was tested using a design-based method. Specifically, we added a third condition to the experiment in which participants would receive a personalized feedback report that had been stripped of all normative general population information. We had also intended to employ a mediator-based method, in which it was predicted that participants in the full personalized normative feedback intervention condition who reported greater reductions in their estimates about how much others gamble between baseline and three-month follow-up would demonstrate more improvement in gambling outcomes at six-month follow-up, compared to respondents in the intervention condition who reported smaller reductions in their perceived gambling norms. However, as will be explained in the Results section, perceived norms regarding others gambling, an error was made in the measurement of perceived norms at baseline, making it unfeasible to conduct this mediator analysis.

## Methods

The protocol for this trial and supporting CONSORT checklist are available as supporting information; see Checklist S1 and Protocol S1.

### Ethics

The study was conducted in compliance with the Helsinki Declaration. Verbal consent was obtained from all participants as the initial contact was by telephone. Interviewers were trained in appropriate ethics procedures and telephone interviews were monitored by a supervisor to ensure adherence to training. This consent procedure and the conduct of the study were approved by the standing ethics review committee of the Centre for Addiction and Mental Health.

### Study Design and Population

This study employed a randomized controlled design with a modified waiting list control. The target population was adult (18 years and over) problem gamblers, encompassing the full range of potential problems from moderate problem gambling to gambling dependence as defined by the Problem Gambling Severity Index, PGSI [23]. Respondents were recruited through a random digit dialing telephone screener of the Ontario population conducted by the Institute for Social Research (ISR), York University. The screener identified current problem gamblers using the PGSI (score of 3 or more), asked a series of questions regarding gambling behaviours and beliefs, and respondent demographic characteristics. These items included the PGSI, the Gambling Cognitions Questionnaire, GCQ, perceptions of other peoples' gambling, and other demographic items (age, sex, education, marital status, occupational status, family income). Following the work of Hodgins and colleagues [5], the primary outcome measures were: a) mean number of dollars lost per month; b) mean days gambled per month; c) greatest dollar amount gambled on any one day; and d) total PGSI score.

To identify those interested in self-help materials, respondents were told that, “the next question asks about self-help services for gamblers that the Centre for Addiction and Mental Health may provide in the future,” and then asked, “If the service was offered for free, would you be interested in receiving a computerized summary that compared your gambling to other Canadians?” At the end of the screener problem gamblers who indicated interest in self-help materials were asked if they would be interested in taking part in another study, “to help us develop and evaluate self-help materials for gamblers.” They were told that the materials and the three-, six- and 12-month follow-up surveys would be mailed to them and that they would be paid $60 for their participation ($20 for the completion of each survey). Respondents who were willing to participate in the further study provided their names and addresses, and were randomly assigned to one of three conditions: 1) the full personalized normative feedback intervention condition; 2) a partial feedback condition that contained all the feedback information provided to those in condition 1 with the exception that all normative feedback content was removed (i.e., no comparisons provided to general population gambling norms); or 3) to the waiting list control condition. Verbal consent was obtained as the initial contact is by telephone. Respondents were allocated to intervention and control conditions using a random number list generated for the study by the principal investigator. No stratification was employed, however, randomization was conducted by block in order to ensure equal number of participants per condition. The personalized feedback for the respondents in the intervention conditions was generated and mailed to them shortly after the telephone interview. Respondents in the waiting list control condition received the full personalized feedback intervention after completion of the six-month follow-up. The design is described as a modified waiting list control method because the wording of the study description ensured that respondents volunteering for the study would not have an expectation that they would receive self-help materials right away. Thus, instead of receiving self-help materials at baseline, respondents in the waiting list control condition were asked to tell us what they think should be included in self-help materials for gamblers.

### Full Personalized Normative Feedback Intervention

The personalized feedback materials start out with a brief statement of the purpose of the report (i.e., “help to give you a picture of your gambling and let you know how your gambling compares with other Canadians”). The person is then provided with a summary of the number of different types of gambling they engage in, along with a comparison of how this total number compares to other Canadians of their sex. Population estimates were derived from the 2002 Canadian Community Health Survey [24]. A list is then provided of all of the gambling activities that the person engaged in at least once a month. For each of the gambling activities listed the person is then provided with a graphical figure that visually demonstrates where their gambling fits in comparison with other Canadians. Including feedback only for gambling activities the person engages in at least once per month ensures that the person only receives normative feedback for gambling activities where they gamble more than the majority of Canadians. The feedback then provides a summary of their PGSI along with a description of their scores (i.e., non-problem gambler, low risk gambler, moderate risk gambler, problem gambler). The feedback continues with a list of the actual problems the respondent reported on the PGSI. The next section comprises of a description of the types of gambling cognitions that the person endorsed on the Gambling Cognitions Questionnaire, a measure of the cognitive distortions the person holds about gambling. For each distorted cognition the person holds (e.g., “I try to figure out what my luckiest numbers are”), a summary about the error of each of these beliefs is provided. These summaries were adapted from a self-help book for problem gamblers [7]. The final element of the feedback is a list of techniques that the person could adopt to lower the risk associated with their gambling. Finally, a comparison is provided of the amount of money the person spent in the past year with the average amount of money spent by Canadians of the same sex. The reader can access a complete version of the Check your Gambling personalized feedback intervention at www.CheckYourGambling.net. An example Final Report of the full normative feedback is contained in Appendix S1.

### Partial Personalized Feedback Intervention

The partial feedback was generated using the same CheckYourGambling.net software and then all normative comparison information was removed.

### Statistical Analyses

Based on the results of our pilot trial and following the convention that studies should be designed to have a statistical power of at least 80%, and that hypotheses be tested using tow-sided tests at the .05 level of significance, a power analysis has estimated a final sample (required after attrition) of 57 respondents per condition. This estimate was obtained for the full personalized feedback and control groups, prior to the addition of a third group, personalized feedback without the normative component. The third condition was added after receipt of funding but before study commencement to examine whether personalized feedback required normative information to be effective. Comparable sample sizes have been used for the three condition design; however a revised power analysis has not been carried out and the initial power analysis for two conditions is presented here. Therefore, assuming a 79% follow-up rate, as was obtained in a self-help intervention study for problem gamblers [5], it was calculated that 217 problem gambling respondents agreeing to participate in the study will have to be recruited at baseline to obtain 171 completed follow-ups (171/0.79) for the two conditions.

Prior to conducting the outcome analyses, gambling data at baseline, three-, six- and 12-month follow-ups were examined for their distributional characteristics. For participants whose follow-up questionnaire was not returned at any of the time points, missing data for the questionnaire not returned was replaced with the corresponding baseline data (analyses were also conducted without replacement of missing data with similar results to those reported here). Gambling variables were positively skewed so they were Winsorized by replacing any outliers beyond three standard deviations with the next highest value (this resulted in gambling variables that approached normal distributional characteristics). Of the four primary outcome variables chosen for this analysis, the analysis investigating possible changes in problem gambling severity as measured by the PGSI was not conducted as there was extensive missing data on the follow-up questionnaires for this scale (a third of the participants listed “Refused” or “Don't Know” for at least one of the nine items on one or more of the follow-up questionnaires).

Analyses were conducted using 3×4 repeated measures ANOVAs. The within subjects variable was time of follow-up (baseline, three-, six-, and twelve-month follow-up). Intervention condition (received full personalized feedback intervention, received partial feedback intervention, control group) was the between subjects variable. A Bonferroni adjustment was applied in order to control for multiple statistical tests (.05/3 = .02; significance level set at *p*<.02).

### Participant Recruitment and Characteristics

A random digit dialing telephone survey of 8015 respondents who spent more than $100 on gambling in the last year was conducted in Ontario between December 2007 and January 2010. Of these 8015 respondents, 766 scored three or more on the PGSI and were asked the core survey. A total of 304 (39.7% of 766) respondents said that they were interested in receiving a computerized summary that compared their gambling to other Canadians if it was offered for free and 209 of these (68.7% of 304) stated that they would be willing to take part in a study to help us develop and evaluate self-help materials for problem gamblers (see Figure S1 for Consort Diagram). Table 1 presents the results of attrition analyses, comparing the demographic and gambling characteristics between those not interested in personalized feedback (n = 462), those interested in feedback but not interested in participating in the follow-up study (n = 95), and those agreeing to take part in the randomized trial (n = 209). Participants who agreed to take part in the randomized trial were more likely to report a household income of less than $30,000 as compared to participants who were not interested in the study (χ^2^ = 19.6, 4 df, *p* = .001; note that this reflected a lower proportion of people refusing to provide household income information in this condition rather than actually having a greater proportion of people with a low family income). In addition, participants who agreed to take part in the trial reported higher PGSI scores at baseline compared to participants who were not interested in personalized feedback interventions, F(2, 763) = 17.3, *p*<.001; Scheffe post hoc test, *p*<.05.

**Table 1:** Attrition analysis for potential respondents screened out prior to consent (n = 766).

Variable	Not interested in personalized feedback(n = 462)	Interested in feedback but not in study(n = 95)	Consented to participate in study(n = 209)	*p*
Mean (SD) Age	49.0 (15.7)	48.4 (14.8)	46.6 (13.9)	N.S.
% Male	56.9	54.7	52.6	N.S.
% Some post-secondary education	50.2	51.6	51.2	N.S.
% Married/Common law	56.5	48.4	55.3	N.S.
% Full/Part-time employed	57.0	60.0	55.8	N.S.
% Family income				
<$30,000	14.9	16.8	24.9	
$30,000 or more	67.7	67.4	68.4	
Don't know/Refused	17.3	15.8	6.7	.001
Mean (SD) PGSI score^a^	5.4 (3.3)	6.4 (4.1)	7.2 (4.8)	.001

N.S. = Not significant, *p*>.05.

a PGSI is the problem gambling severity index. Only participants with scores of 3 or more, indicating current hazardous gambling were included in the attrition analyses.

Of the 209 participants in the randomized trial, 84.2% (n = 176) provided follow-up data for at least one of the follow-up points. Specific follow-up rates at each time point were: 3-month follow-up = 77% (n = 161); 6-month follow-up = 75.1% (n = 157); 12-month follow-up = 69.9% (n = 146). There were no significant (*p*>.05) differences in attrition rates between the different experimental conditions. Table 2 presents comparisons of demographic and gambling characteristics between participants who completed at least one follow-up with those participants who did not return any follow-up questionnaires. The only difference observed was that participants who completed at least one follow-up were older than participants who did not return any of the follow-ups. Further, bivariate analyses were conducted to compare demographic and gambling characteristics between participants in the three experimental conditions at baseline. There were no significant differences between conditions (*p*>.05).

**Table 2:** Comparison of participants with at least one follow-up completed (n = 176) to those who did not complete at least one follow-up (n = 33).

Variable	Did not complete a follow-up(n = 33)	Completed at least one follow-up(n = 176)	*P*
Mean (SD) Age	41.2 (15.6)	47.6 (13.4)	.02
% Male	60.6	51.1	N.S.
% Some post-secondary education	42.4	52.8	N.S.
% Married/Common law	45.5	57.1	N.S.
% Full/Part-time employed	63.6	54.3	N.S.
% Family income			
<$30,000	24.2	25.0	
$30,000 or more	66.7	68.8	
Don't know/Refused	9.1	6.3	N.S.
PGSI score^a^	6.8 (4.1)	7.3 (4.9)	N.S.

N.S. = Not significant, *p*>.05.

a PGSI is the problem gambling severity index. Only participants with scores of 3 or more, indicating current hazardous gambling were included in the attrition analyses.

## Results

Means for the three primary gambling outcome variables, amount of money spent on gambling in the past 30 days, number of days in which gambled out of the past 30, and the most money spent on gambling in one day are displayed in Table 3. A 3×4 repeated measures ANOVA for the outcome variable, amount of money spent, found a main effect of time of follow-up, F(3, 195) = 3.8, *p*<.02, but not significant interaction between Time of follow-up and intervention condition indicating all conditions reported reducing the amount of money they spent from baseline to follow-up but that there was no differential impact of the interventions on this reduction.

**Table 3:** Mean (*SD*) gambling variables at baseline, three-, six-month, and 12-month follow-up by study condition (n = 209).

	Feedback condition			
Time	Full feedback(n = 70)	Partial feedback(n = 70)	Waiting list^a^(n = 69)	*p*
	**Total Dollars Spent on Betting Past 30 Days**			
Baseline	471.1 (631.5)	569.2 (690.0)	407.0 (599.5)	
3-month	432.0 (514.3)	481.4 (542.3)	334.8 (418.6)	
6-month	361.8 (476.9)	412.8 (550.0)	327.2 (420.7)	
12-month	378.4 (476.0)	366.0 (503.0)	348.8 (468.3)	T
	**Number of Days Gambled in Past 30 Days**			
Baseline	10.9 (9.0)	10.2 (8.5)	9.0 (6.9)	
3-month	11.3 (8.6)	9.0 (7.8)	8.9 (6.2)	
6-month	9.3 (8.3)	8.6 (7.5)	8.6 (6.6)	
12-month	10.9 (9.1)	7.2 (7.3)	9.7 (7.6)	T X I
	**Largest Amount Spent on Gambling on Any Day**			
Baseline	486.7 (625.4)	483.9 (584.0)	385.3 (519.7)	
3-month	331.8 (446.1)	342.8 (418.0)	235.9 (289.6)	
6-month	286.9 (378.0)	281.6 (371.6)	223.7 (280.9)	
12-month	263.2 (333.7)	285.4 (335.5)	228.2 (269.4)	T

a Participants in waiting list control group sent full normative feedback intervention after 6-month follow-up.

T = Main effect of time of follow-up, *p*<.02. T X I = Interaction between time of follow-up and intervention condition, *p*<.02.

A separate 3×4 repeated measures ANOVA comparing number of days gambling in the past 30 days at baseline, three-, six-, and 12-month follow-ups between intervention conditions and problem gambling status at baseline found there was a significant Time X Intervention interaction, F (6, 358) = 2.9, *p*<.01. Post-hoc analyses revealed that, compared to participants in the waiting list control condition, and in the full feedback condition, participants in the partial feedback condition displayed a significant reduction in the number of days gambled from baseline to the twelve-month follow-up (*p*<.05). Figure S2 displays the results of this significant time by intervention interaction.

Finally, a 3×4 repeated measures ANOVA for the outcome variable (largest amount of money spent on one day) found a main effect of time of follow-up, F(3, 199) = 13.1, *p*<.001, but not significant interaction between Time of follow-up and intervention condition indicating all conditions reported reducing the largest amount of money they spent from baseline to follow-up but that there was no differential impact of the interventions on this reduction.

### Perceived norms regarding others gambling

An error was made in the wording of the perceived norms questions when constructing the baseline telephone interview. Unlike the follow-up questionnaire, where participants are asked to estimate how much others of the same age and sex gambled (separate questions for number of days in past 30 days, amount of money spent in past 30 days and largest amount spent on one day), the baseline telephone survey asked these same questions but worded to ask about ‘people like you’ rather than ‘of the same age and sex.’ This error makes the interpretation of any possible changes in perceived norms resulting from receiving the full personalized feedback report after the baseline interview problematic. However, we can use data from participants in the waiting list control group who were given the full personalized feedback report after sending in their 6-month follow-up and compare any changes in perceptions of others gambling to those in the partial feedback condition (who never receive any normative information). As with the outcome variables used to assess levels of gambling in the participants, the variables employed to test changes in perceptions of other's gambling were examined for outliers and Winsorized to normalize the distribution. However, missing data were not replaced and the significance level was not adjusted to reflect multiple statistical tests because of the exploratory nature of these analyses.

Means and standard deviations for the perception of others gambling variables are displayed in Table 4 below. A repeated measures 2×2 ANOVA with time of follow-up (6-month versus 12-month follow-up) as the within subjects variable and experimental condition (waiting list control versus partial feedback) as the between subjects variable found a time by interventions interaction on perceptions of how much others of the same age and sex spent on gambling in the past 30 days, F(1,83) = 3.9, *p* = .05. Inspections of the means indicated that participants in the waiting list control (who received the full feedback intervention after the 6-month follow-up) reduced their perceptions of how much others spend on gambling by the 12-month follow-up while participants in the partial feedback condition did not reduce their perceptions of how much others spend on gambling.

**Table 4:** Mean (*SD*) perceptions of other's gambling variables at six-month, and 12-month follow-up by study condition (n = 87).

	Feedback condition		
Time	Waiting list control^a^(n = 40)	Partial feedback(n = 47)	*p*
	**Others Total Dollars Spent on Betting Past 30 Days**		
6-month	206.7 (204.4)	258.4 (314.8)	
12-month	149.2 (136.4)	298.1 (329.1)	T X I
	**Others Number of Days Gambled in Past 30 Days**		
6-month	8.6 (5.5)	8.7 (6.2)	
12-month	8.4 (4.5)	7.0 (5.0)	
	**Others Largest Amount Spent on Gambling on Any Day**		
6-month	232.8 (298.3)	232.2 (260.0)	
12-month	164.2 (189.9)	219.9 (256.9)	T

a Participants in waiting list control group sent full feedback intervention after 6-month follow-up.

T X I = Interaction between time of follow-up and intervention condition, *p* = .05.

T = Main effect of time, *p*<.03.

A separate 2×2 repeated measures ANOVA found no significant (*p*>.05) main or interaction effects for the variable, perception of how many days in the past 30 days other people gamble. Finally, a 2×2 repeated measures ANOVA examining perceptions of the largest amount others of the same sex spend on gambling found a main effect of time of follow-up, F(1,84) = 5.2, *p*<.03, but no significant (*p*>.05) interaction between time and condition.

## Discussion

The results of this randomized trial were unexpected. We had predicted that the full personalized feedback intervention, which included extensive normative feedback, would have an impact on levels of gambling at follow-up. The partial feedback intervention (with no normative feedback) was added in order to see whether it was the norms that were important in leading to change. If we saw any impact of either of the interventions, the expectation was that the full normative feedback would be more likely to have an impact than the partial feedback. Further, our pilot trial [22] had found some initial evidence that the full normative feedback intervention could reduce levels of gambling and similar interventions targeting problem drinking also had demonstrated that personalized feedback incorporating norms were efficacious. Instead, what was observed in this trial were some reductions in levels of gambling in participants who received the partial feedback (at least in number of days gambled) but no significant impact of the full personalized normative feedback intervention, as compared to participants in the waiting list control group.

While the analyses did include a Bonferroni correction to account for multiple tests, it is a concern that only one of the three outcome measures, number of days gambled in the past 30 days, showed an impact of the intervention. The other two variables, total dollars spent in the past 30 days, and largest amount spent on gambling in the past 30 days, showed no effect of condition but did display reductions over time. Such reductions, even without an intervention, are a common occurrence in a trial of this type and could reflect a regression to the mean or an actual natural history improvement in levels of gambling [25]. It is also interesting to observe the pattern of results (even if not significant). It appears that participants in the waiting list condition displayed an initial reduction in gambling between baseline and three-months but then no further changes, whereas those in the intervention conditions displayed some trend towards continued reductions over the three follow-up points. This initial reduction could potentially also represent an impact of the assessment as administration of questionnaires has, in itself, been shown to change behaviour [26], [27]. However, it is important to note that examination of the pattern of results, while informative, can only be taken as speculation given that the current trial was not designed to test hypotheses such as the impact of receiving an assessment.

While these results are interesting, we are left with the challenge of trying to interpret them. We are left with the question as to what the ‘active ingredient’ is if it is not normative feedback. It could be that increasing salience about the amount a person gambles (as discussed in the introduction) is the driving force behind the changes observed, although, if this is the case then we would expect that reductions would have been observed in both feedback conditions. Or, it could be other elements of the feedback, such as the summary of the severity of problems, or the section highlighting erroneous cognitions. However, it is impossible to do more than speculate on these possibilities in the current study as further research partitioning the different elements of the intervention would be needed in order to adequately test for active ingredients in personalized feedback interventions.

There are a number of possibilities as to why the full personalized normative feedback did not result in significant reductions in gambling while the partial one did. The first, and usually the most important to keep in mind, is that these findings could be due to chance. No single randomized trial should be taken as proof of the impact of an intervention without consistent replication of the findings in at least one other randomized trial (or preferably, several). This feedback intervention was subjected to a pilot trial which did show some evidence of impact when the normative information was included. The current trial showed an impact of the intervention but only when the normative information was not included. It is reasonable to conclude from these results that there is some sort of impact that can result from receiving some version of this intervention, but the best version of the materials and the size of the impact is not clear as of yet.

Another explanation of the differences observed between the pilot trial and this trial was that the samples employed in the study were very different. The pilot trial was an add on to a study that was designed to develop typologies of gambling [28]. Those recruited, while not seeking treatment, were willing to show up for an extended face-to-face interview at a facility that provides gambling treatment. Further, participants in the pilot trial had an average PGSI score of 15, indicating substantial gambling problems. The current study recruited participants from a random digit dialing telephone survey of Ontario adults and attempted to recruit participants with PGSI score of 3 or more (average PGSI score of 7). These are no doubt very different participant populations and some aspect of this difference could be driving different reactions to different components of this intervention. As an example, while normative feedback is only provided when the participant actually gambles more than that reported in the general population, it is possible that the normative feedback may be more salient to people with more severe gambling problems. However, it is difficult to ascertain what differences may be significant from these studies.

Further, it is possible that the lack of impact observed with the full personalized feedback could be an indication of the difficulties we had in creating adequate normative feedback for problem gamblers. Good normative feedback requires high quality general population data on gambling. To do this, a very large data set is needed because heavy gambling is relatively infrequent and good normative feedback seems to benefit from providing sex and age specific norms (the current feedback could only provide sex specific norms because the general population data set employed, while comprising of more than 32,000 participants, was still not large enough to generate stable population estimates by age and sex). In addition, gambling may prove more difficult to generate easily interpretable normative feedback than norms interventions for drinking because there are many different types of gambling and it is unclear whether the norms generated need to be specific to the type of gambling under discussion. Finally, the norms were gambling activity specific, while the outcome measures were global summary measures of all gambling activities. This could make the outcome measures less sensitive to the impact of the norms provided.

It is also possible that normative feedback is just not relevant to gamblers. We did find some limited evidence that providing norms did lead to recipients modifying their estimates of how much others of the same age and sex gamble. However, correcting this normative misperception may not mediate changes in actual levels of gambling in the same way that correcting normative misperceptions in drinkers appears to cause reductions in the amounts that people drink.

### Summary and Future Directions

This randomized controlled trial found some limited evidence that one version of a gambling personalized feedback intervention could motivate reductions in gambling. Combined with the results of the pilot study, which also found some impact of this brief intervention (albeit with a different version), there is a reasonable start for a research base to indicate that personalized feedback interventions are helpful to motivate reductions in problem gamblers. It is hoped that these studies will be joined by research conducted by other research teams on the efficacy of these brief interventions so that an adequate research base can be established in this area.

At this point it can be concluded that personalized feedback interventions are well received by problem gamblers and that the materials may be helpful at reducing their gambling. Realistically, it can be expected that the personalized feedback intervention will have a limited, short term impact on the severity of participants' problem gambling due to the intervention being a brief screener. However, Internet-based intervention tools may offer an easy to access and non-threatening portal to motivate participants to seek further help online or in person. The use of the online screeners remains to be studied and will be an important indicator for the long term benefits of promoting screeners for problem gamblers.

## Supporting Information

Figure S1 **CONSORT diagram of participant recruitment.**
(TIF)

Figure S2 **Mean number of days gambled in the past 30 for participants in the three conditions.**
(TIF)

Protocol S1 **Research protocol as it was funded.**
(DOC)

Checklist S1 **Consort checklist.**
(DOC)

Appendix S1 **Example of materials sent to participants in Full Personalized Feedback Condition.**
(TIF)

## References

[1] Cunningham JA (2005). Little use of treatment among problem gamblers.. Psychiatric Services.

[2] Hodgins DC, el-Guebaly N (2000). Natural and treatment-assisted recovery from gambling problems: a comparison of resolved and active gamblers.. Addiction.

[3] Rockloff MJ, Schofield G (2004). Factor analysis of barriers to treatment for problem gambling.. Journal of Gambling Studies.

[4] Hodgins DC, Currie S, el-Guebaly N, Peden N (2004). Brief motivational treatment for problem gambling: a 24-month follow-up.. Psychology of Addictive Behaviors.

[5] Hodgins DC, Currie SR, el-Guebaly N (2001). Motivational enhancement and self-help treatments for problem gambling.. Journal of Consulting and Clinical Psychology.

[6] Hodgins DC, Makarchuk K (1997). Becoming a winner: Defeating problem gambling.

[7] Toneatto T, Kosky B, Leo GI (2003). How to quit or reduce your gambling: A personal workbook.

[8] Miller WR, Rollnick S (1991). Motivational Interviewing: Preparing People to Change Addictive Behavior.

[9] Agostinelli G, Brown JM, Miller WR (1995). Effects of normative feedback on consumption among heavy drinking college students.. Journal of Drug Education.

[10] Cunningham JA, Koski-Jännes A, Wild TC, Cordingley J (2002). Treating alcohol problems with self-help materials: A population study.. Journal of Studies on Alcohol.

[11] Miller WR, Sovereign RG, Krege B (1988). Motivational interviewing with problem drinkers: II. The Drinker's Check-up as a preventive intervention.. Behavioural Psychotherapy.

[12] Borsari B, Carey KB (2000). Effects of a brief motivational intervention with college student drinkers.. Journal of Consulting and Clinical Psychology.

[13] Murphy JG, Duchnick JJ, Vuchinich RE, Davison JW, Karg RS (2001). Relative efficacy of a brief motivational intervention for college student drinkers.. Psychology of Addictive Behaviors.

[14] Baer JS, Kivlahan DR, Blume AW, McKnight P, Marlatt GA (2001). Brief intervention for heavy-drinking college students: 4-year follow-up and natural history.. American Journal of Public Health.

[15] Curry SJ, Wagner EH, Grothaus LC (1991). Evaluation of intrinsic and extrinsic motivation interventions with a self-help smoking cessation program.. Journal of Consulting and Clinical Psychology.

[16] Curry SJ, Louie D, Grothaus L, Wagner EH (1992). Written personalized feedback and confidence in smoking cessation.. Psychology of Addictive Behaviors.

[17] Agostinelli G, Miller WR (1994). Drinking and thinking: How does personal drinking affect judgments of prevalence and risk.. Journal of Studies on Alcohol.

[18] Sanchez-Craig M, Davila R, Cooper G (1996). A self-help approach for high-risk drinking: Effect of an initial assessment.. Journal of Consulting and Clinical Psychology.

[19] Spivak K, Sanchez-Craig M, Davila R (1994). Assisting problem drinkers to change on their own: Effect of specific and non-specific advice.. Addiction.

[20] Larimer ME, Neighbors C (2003). Normative misperception and the impact of descriptive and injunctive norms on college student gambling.. Psychology of Addictive Behaviors.

[21] Takushi RY, Neighbors C, Larimer ME, Lostutter TW, Cronce JM (2004). Indicated prevention of problem gambling among college students.. Journal of Gambling Studies.

[22] Cunningham JA, Hodgins DC, Toneatto T, Rai A, Cordingley J (2009). Pilot study of a personalized feedback intervention for problem gamblers.. Behavior Therapy.

[23] Ferris J, Wynne H (2001). The Canadian Problem Gambling Index: Final Report..

[24] Statistics Canada (2003). Canadian Community Health Survey: Mental Health and Well-being, 2002.

[25] Cunningham JA (2006). Regression to the mean: what does it mean?. Alcohol and Alcoholism.

[26] McCambridge J, Day M (2008). Randomized controlled trial of the effects of completing the Alcohol Use Disorders Identification Test questionnaire on self-reported hazardous drinking.. Addiction.

[27] Kypri K, Langley JD, Saunders JB, Cashell-Smith ML (2007). Assessment may conceal therapeutic benefit: findings from a randomized controlled trial for hazardous drinking.. Addiction.

[28] Toneatto T, Turner NE, Zack M, Farvolden P, Millar G (2007). The heterogeneity of problem gambling: An analysis of gambling sub-types.. Ontario Problem Gambling Research Centre.

