# Supplementary figures and images for: A Randomized Controlled Trial of a Personalized Feedback Intervention for Problem Gamblers
Source: PLoS One. 2012 Feb 14;7(2):e31586. doi: 10.1371/journal.pone.0031586 (PMC3279405; doi:10.1371/journal.pone.0031586)

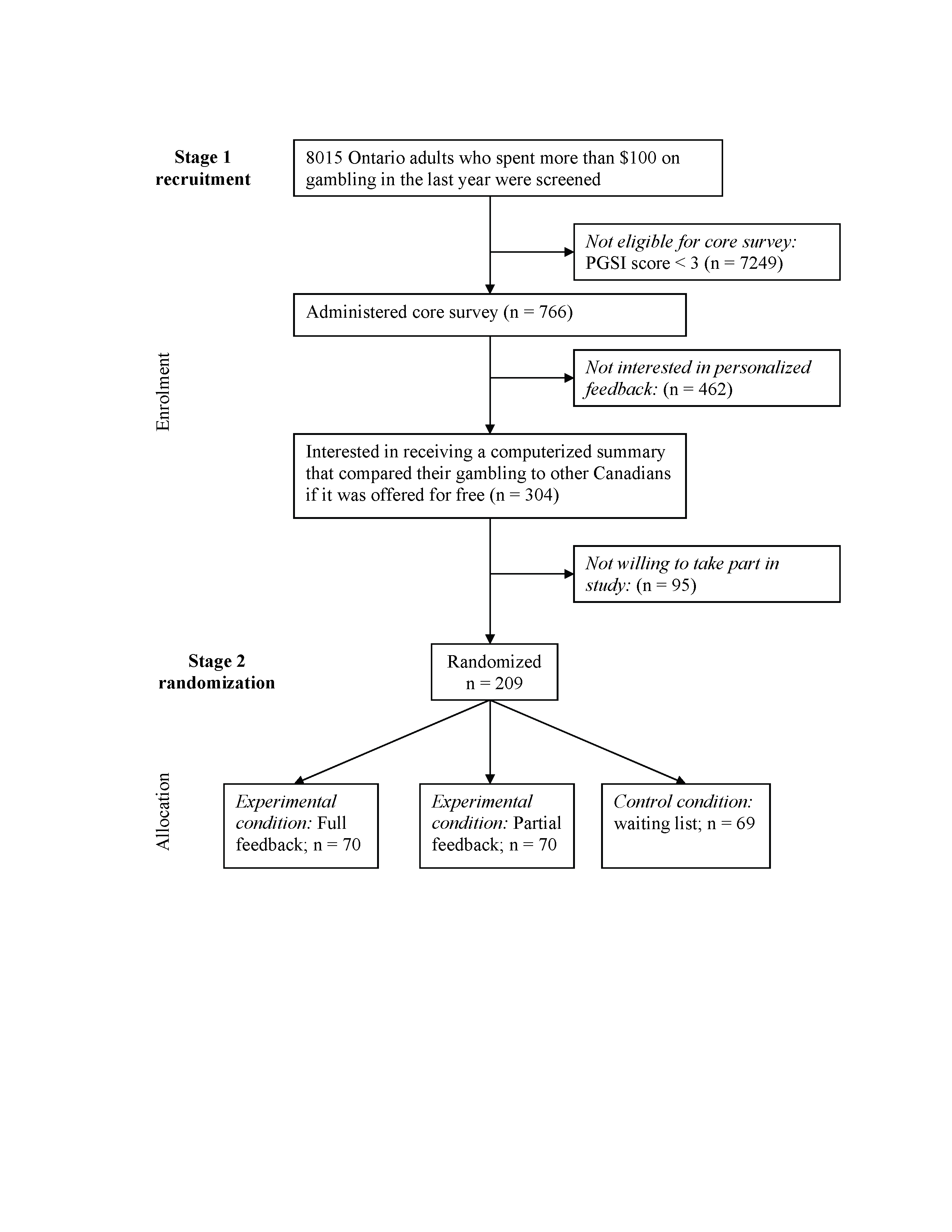

Supplement: Figure S1 — CONSORT diagram of participant recruitment. (TIF) [file pone.0031586.s001.tif]

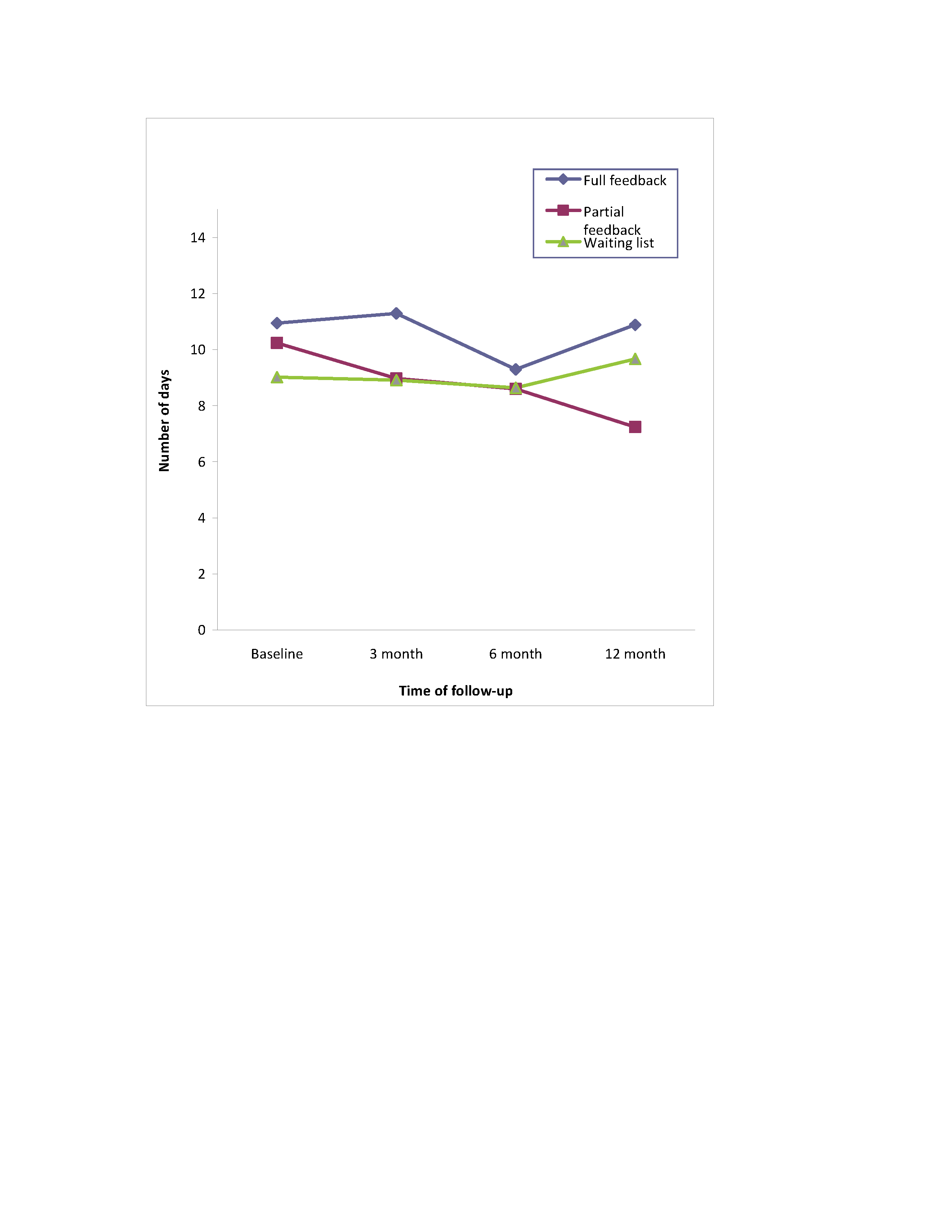

Supplement: Figure S2 — Mean number of days gambled in the past 30 for participants in the three conditions. (TIF) [file pone.0031586.s002.tif]

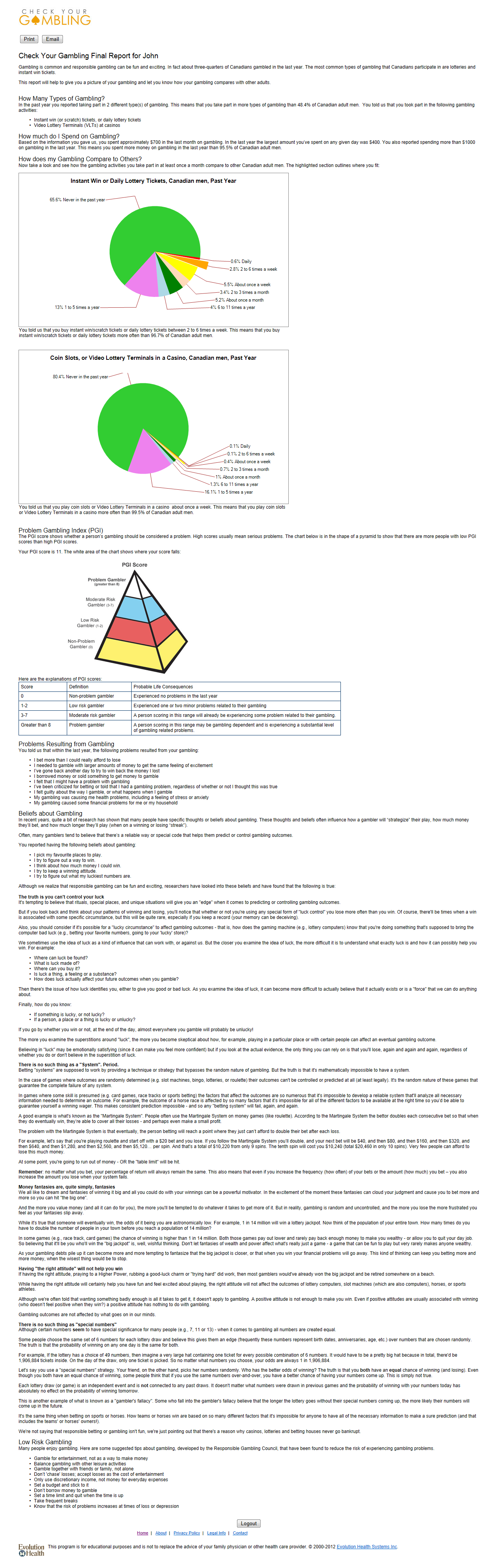

Supplement: Appendix S1 — Example of materials sent to participants in Full Personalized Feedback Condition. (TIF) [file pone.0031586.s005.tif]
