# Supplementary material for: A Randomized Controlled Trial of a Personalized Feedback Intervention for Problem Gamblers
Source: PLoS One. 2012 Feb 14;7(2):e31586. doi: 10.1371/journal.pone.0031586 (PMC3279405; doi:10.1371/journal.pone.0031586)
Supplement: Protocol S1 — Research protocol as it was funded. (DOC) [file pone.0031586.s003.doc]

**Protocol**

**Title:** A personalized feedback intervention for problem gamblers

**Sponsoring organization:** Centre for Addiction and Mental Health

**Status of sponsoring organization:** Hospital

**Principal Investigator:**  Dr. John A. Cunningham

Institutional Affiliation: Centre for Addiction and Mental Health

Position: Senior Scientist

**Co-Investigator:** Dr. David C. Hodgins

Institutional Affiliation: University of Calgary

Position: Professor

**Co-Investigator:**  Dr. Tony Toneatto

Institutional Affiliation: Centre for Addiction and Mental Health

Position: Senior Scientist

**Abstract** - Include an abstract of no more than 500 words for the research project;

Only about one in ten gamblers with a lifetime diagnosis of gambling dependence will ever seek treatment (Cunningham, 2005). Many of these problem gamblers are unwilling to access treatment, often because of stigma, embarrassment or a desire to handle their problems on their own (Hodgins & el-Guebaly, 2000; Rockloff & Schofield, 2004). These problem gamblers can be helped. Research has demonstrated the effectiveness of self-help interventions for gambling problems (Hodgins, Currie, el-Guebaly, & Peden, 2004; Hodgins, Currie, & el-Guebaly, 2001). This area deserves more attention because it addresses a cost-effective means of helping problem gamblers without requiring them to come to treatment. The aim of self-help interventions is to help problem gamblers where they are, thus circumventing many of the barriers associated with traditional treatment.

One promising self-help intervention for problem gamblers is personalized feedback. Such interventions have shown consistently positive results with other addictive behaviours. We have conducted a pilot test of personalized feedback materials for gamblers with positive results (see literature review below). Given these promising findings, the proposed research will evaluate the sustained efficacy of a personalized feedback intervention for problem gamblers.

**Research Topic** - Describe the purpose / rationale for the research, and how this project will extend existing knowledge and make a significant contribution to the field;

The proposed research will evaluate the sustained efficacy of a personalized feedback intervention for problem gamblers. As will be outlined below, personalized feedback is thought to be effective as an intervention because this feedback can correct the common misperception held by many problem gamblers that others gamble as much or more than they do (i.e., correcting the normative fallacy). This proposal will include a test of the potential mediating role of correcting normative estimates. Further, the recruitment methods proposed (a general population telephone screener) will allow the evaluation of these materials with problem gamblers from all regions of Ontario. Finally, the proposed study would include gamblers who have moderate gambling problems as well as those with more severe problems because personalized feedback materials have been found to be helpful for a range of severity of problems in other addictive behaviours. It is hypothesized that: **Hypothesis 1:** Problem gamblers who receive the personalized feedback intervention will reduce their gambling more than problem gamblers who do not receive any intervention (waiting list control condition) by a six-month follow-up. **Hypothesis 2:** The effect of the intervention will be sustained through the 12-month follow-up. **Hypothesis 3 (mediator hypothesis):** Respondents in the intervention condition who report greater reductions in their estimates about how much others gamble between baseline and three-month follow-up will demonstrate more improvement in gambling outcomes at six-month follow-up, compared to respondents in the intervention condition who report smaller reductions in their perceived gambling norms.

The proposed research trial will result in the continued development and evaluation of a new intervention for problem gamblers in Ontario. As has been indicated by the efficacy of personalized feedback for other addictive behaviours and from our pilot study, this personalized feedback intervention has great potential to reduce problem gambling. This research is important because it holds promise to provide another means for helping problem gamblers in Ontario, whether they access treatment or not. The development of effective treatments for problem gamblers has been identified as a research priority by the Ontario Problem Gambling Research Centre (OPGRC). In addition, the OPGRC is particularly interested in issues of barriers to treatment and treatment engagement. The personalized feedback intervention is specifically designed to engage problem gamblers outside of traditional treatment settings, helping them where they are and also, promoting interest in accessing formal treatment. Finally, the personalized feedback intervention has the advantage of being compatible with existing gambling services – it can be used in standard treatment settings, in conjunction with other self-help interventions, and as a stand-alone intervention to reduce the harm associated with problem gambling.

**Literature Review** - Briefly discuss the present state of knowledge in the field (literature);

To-date, research on self-help interventions for problem gamblers has focused on evaluating the efficacy of materials that guide individuals through a series of exercises to help them deal with their gambling. In effect, such interventions are providing standard treatment in a book format (Hodgins & Makarchuk, 1997; Toneatto, Kosky, & Leo, 2003). While this method is effective, are there other means of providing help for problem gamblers not willing to seek treatment? A self-help method found effective with other addictive behaviours is personalized feedback summaries. These brief interventions would allow problem gamblers to evaluate their own gambling behaviour, providing them with summaries of their own gambling activities and comparing their gambling to that of others in the general population. This normative feedback technique is one of the central elements of Motivational Interviewing (Miller & Rollnick, 1991) and has been consistently found to have an impact on a variety of different substance use concerns. Personalized feedback has been found to promote behaviour change in drinkers (Agostinelli, Brown, & Miller, 1995; Baer, Kivlahan, Blume, McKnight, & Marlatt, 2001; Borsari & Carey, 2000; Cunningham, Koski-Jännes, Wild, & Cordingley, 2002; Miller, Sovereign, & Krege, 1988; Murphy et al., 2001) and smokers (Curry, Louie, Grothaus, & Wagner, 1992; Curry, Wagner, & Grothaus, 1991). In drinkers, normative feedback is theorized to promote changes in alcohol use because many heavy drinkers overestimate the consumption of others. Consequently, normative feedback acts as a powerful source of social comparison, motivating heavy drinkers to re-evaluate their consumption patterns (Agostinelli & Miller, 1994). The same motivational principles are hypothesized to promote change in other addictive behaviours (Miller & Rollnick, 1991).

Dr. Sanchez-Craig and colleagues (Sanchez-Craig, Davila, & Cooper, 1996; Spivak, Sanchez-Craig, & Davila, 1994) speculated on another reason why personalized feedback interventions might have an impact after finding that providing personalized feedback to problem drinkers in addition to a self-help book resulted in a greater reduction in drinking (as compared to a self-help book alone condition). They hypothesized that one of the reasons that the feedback had an impact was that it made the amount the person drank explicit. Thus, for gamblers, personalized feedback might also work because it makes the amount the person gambles explicit. We propose to test whether our personalized feedback intervention has an effect through correcting normative misperceptions by including a mediator hypothesis in this research trial. In this way, we will be able to determine whether any reductions in gambling are just due to making the amount people gamble explicit or because we are correcting normative misperceptions.

Is there any evidence to indicate that personalized feedback interventions would work with gamblers?

Gamblers often overestimate how much others are gambling (Larimer & Neighbors, 2003). The existence of this normative fallacy is the key condition that is required for personalized feedback to work. It is predicted that, as for problem drinkers and smokers, when presented with normative information showing that most people gamble less than they do, gamblers will be motivated to re-evaluate their gambling behaviour and reduce the amount they gamble. In fact, several authors have posited that personalized feedback interventions would work for problem gamblers (Larimer & Neighbors, 2003; Takushi et al., 2004). In addition, a pilot study conducted by this research team has provided positive evidence of the potential of this brief intervention (Cunningham, Hodgins, Toneatto, Rai, & Cordingley, under review). In this pilot, 61 respondents were recruited from an ongoing gambling research study to take part in another study to help us “develop and evaluate self-help materials for gamblers.” Respondents who agreed to participate were randomly assigned to receive a personalized feedback summary or to a waiting list control. At three-month follow-up (80.3% follow-up rate, N = 49), after controlling for baseline demographic characteristics and gambling severity, respondents in the feedback condition displayed some evidence that they were spending less money on gambling as compared to those in the control condition (*p* < .05). See Table 1 below for a summary of the results. As can be observed, participants in the intervention group were losing 75% less overall than the control group and their maximum amount gambled on average was 50% smaller. If the level of these reductions can be replicated in the proposed trial, these changes represent clinically meaningful reductions in addition to being statistically significant.

**Table 1: Estimated mean^a^** gambling expenditures and CPGI scores for respondents in the personalized feedback and control conditions at three-month follow-up

|  |  |  |
| --- | --- | --- |
|  | Feedback | **Control** |
|  | **(n = 24)** | **(n = 25)** |
|  |  |  |
|  |  |  |
| Mean (SE) Total amount spent | 563.5 (445.5) | 2267.1 (445.5) |
|  |  |  |
| Mean (SE) Maximum spent | 184.5 (99.8) | 391.2 (99.8) |
|  |  |  |
| Mean (SE) CPGI at follow-up | 11.0 (0.8) | 12.6 (0.9) |
|  |  |  |

^a^ Means are estimated after controlling for demographic characteristics and baseline CPGI scores.

In addition to looking at changes in amount of gambling, the pilot test explored respondents’ reactions to the feedback materials. Ratings of the usefulness of the feedback summary were positive and almost all recipients (96%) recommended that they be made available to other gamblers interested in evaluating or modifying their gambling. Given these promising pilot results, a full-scale evaluation of these personalized feedback materials would appear justified.

*Summary of the Content of the Personalized Feedback Intervention*

The appendix to this proposal contains a complete copy of the feedback materials used in the pilot trial. The personalized feedback materials start out with a brief statement of the purpose of the report (“help to give you a picture of your gambling and let you know how your gambling compares with other Canadians”). The person is then provided with a summary of the number of different types of gambling they engage in, along with a comparison of how this total number compares to other Canadians of their sex (population estimates from the 2002 Canadian Community Health Survey on Mental Health and Well-being were used for this purpose, CCHS,Statistics Canada, 2003). A list is then provided of all of the gambling activities that the person engaged in at least once a month. For each of the gambling activities listed, the person is then provided with a graphical figure that visually demonstrates where their gambling fits in comparison with other Canadians (the white segment of the graph).

The feedback then provides a summary of their Canadian Problem Gambling Index score (CPGI, Ferris & Wynne, 2001) along with a description of what their score means (non-problem gambler, low risk gambler, moderate risk gambler, problem gambler). The feedback continues with a list of the actual problems the respondent reported on the CPGI. The next section comprises a description of the types of gambling cognitions that the person endorsed on the Gambling Cognitions Questionnaire (GCQ, Toneatto, 1999), a measure of the cognitive distortions the person holds about gambling. For each distorted cognition the person holds (e.g., “I try to figure out what my luckiest numbers are”), a summary about the error of each of these beliefs is provided. These summaries were adapted from a self-help book for problem gamblers (Toneatto, Kosky, & Leo, 2003). The final element of the feedback is a list of techniques that the person could adopt to lower the risk associated with their gambling.

A further element, a comparison of the amount of money the person spent in the past year (and percent of total income) with the average amount spent by Canadians of the same sex, was also developed. However, as information on amount of income spent on gambling was not collected as part of the research project this pilot study was appended to this feedback was not provided to respondents. This feedback element will be included in the version of the materials to be evaluated in this full research trial.

**Research Design** - Identify the type of research design, describe the problem statement, hypotheses, or research questions that will guide the research; describe the methodology (sampling, data collection and analysis, instrumentation, validity and reliability, limitations/delimitations);

A randomized controlled trial with a modified waiting list control method will be employed to test the hypotheses. The target population for this research will be adult (18 years and over) problem gamblers, encompassing the full range of potential problems from moderate problem gambling to gambling dependence as defined by the Canadian Problem Gambling Index (Ferris & Wynne, 2001). Respondents will be recruited through a random digit dialing telephone screener of the Ontario population. The screener will identify current problem gamblers using the CPGI and will ask a series of questions regarding gambling behaviours and beliefs, and respondent demographic characteristics that are relevant to the generation of the personalized feedback intervention summaries. To identify those interested in self-help materials, respondents will be told that, “the next question asks about self-help services for gamblers that the Centre for Addiction and Mental Health may provide in the future,” and then ask, “if the service was offered for free, would you be interested in receiving a computerized summary that compared your gambling to other Canadians?” At the end of the screener, problem gamblers who are interested in self-help materials will be asked if they are interested in taking part in another study to “help us develop and evaluate self-help materials for gamblers.” They would be told that the materials and the three-, six- and 12-month follow-up surveys would be mailed to them and that they would be paid $60 for their participation ($20 for the completion of each survey). Respondents who are interested will provide their names and addresses. Respondents will then be randomly assigned to the personalized feedback intervention condition or to a waiting list control condition. Respondents in the waiting list control condition will receive the personalized feedback intervention after the six-month follow-up. The design is described as a modified waiting list control method because the wording of the study description will ensure that respondents volunteering for the study will not have the expectation that they will necessarily receive self-help materials right away. Thus, instead of receiving self-help materials at baseline, respondents in the waiting list control group will be asked to tell us what they think should be included in self-help materials for gamblers.

*Hypotheses:* Hypothesis 1: Problem gamblers who receive the personalized feedback intervention will reduce their gambling more than problem gamblers who do not receive any intervention (waiting list control condition) by a six-month follow-up. Hypothesis 2: The effect of the intervention will be sustained through the 12-month follow-up. Hypothesis 3 (mediator hypothesis): Respondents in the intervention condition who report greater reductions in their estimates about how much others gamble between baseline and three-month follow-up will demonstrate more improvement in gambling outcomes at six-month follow-up, compared to respondents in the intervention condition who report smaller reductions in their perceived gambling norms.

*Measures of Gambling – Baseline and Follow-up Surveys:* Following the work of Hodgins and colleagues (2001), the primary outcome measures will be: a) mean number of dollars lost per month; b) mean days gambled per month; c) greatest dollar amount gambled on any one day; and d) total CPGI score.

*Process Measures:* In order to test hypothesis 3, that reductions in gambling are mediated by respondents correcting their estimates of how much others gamble, we will measure respondents’ beliefs about how much other Canadians gamble at baseline and on the three-month follow-up (asking about males for male respondents and females for female respondents). Items will be adopted from the work of Larimer and colleagues (2003) and modified to ask about gambling in the general population rather than gambling among college students.

*Analysis Plan:* Three- and six-month gambling outcomes will be compared between conditions using hierarchical multiple regression (specifically, Analyses of Partial Variance, APV, Cohen & Cohen, 1983). Baseline scores on each primary outcome variable will be entered in the first step of each analysis, producing residualized change scores, i.e., change in gambling use from pre- to post-intervention, adjusted for participants’ baseline gambling behaviour. Secondary analyses will explore the stability of change at twelve-month follow-up. The role of perceived norms as a moderator (hypothesis 3) will be tested by adding an interaction term to the regression equation predicting six-month gambling outcomes. This interaction term will consist of the multiplication of the respondents’ experimental condition (dummy coded as 0 = control condition, 1 = experimental condition) by the difference between respondents’ estimates of how much others gamble measured at baseline and at three-month follow-up. If perceived norms act as a mediator then this interaction term will be a significant predictor of outcomes at six-month follow-up. The final set of analyses to be conducted will look for sex differences in the impact of the intervention materials. While none are predicted, it is important to explore this issue. As with the mediator analysis described above, the moderating role of respondents’ sex will be tested by adding an interaction term between experimental condition and respondent sex to the regression equation to evaluate whether there are sex differences in the degree of impact of the personalized feedback materials.

*Power Analysis:* A power analysis using the results from our pilot study has estimated a final sample (required after attrition) of 57 respondents per condition or 114 for the entire study. Assuming a 79% follow-up rate (as was obtained in a self-help intervention study for problem gamblers, Hodgins, Currie, & el-Guebaly, 2001), 144 problem gambling respondents agreeing to participate in the study will have to be recruited at baseline to obtain 114 completed follow-ups (114 / 0.79). Other pilot work conducted by the author with general population samples has indicated that 37.5% of problem gamblers are interested in receiving personalized feedback materials and that roughly half of eligible respondents will agree to participate in the additional research study (these estimates are derived from an ongoing telephone survey of the Ontario general population exploring barriers to gambling treatment conducted by this research team). This means that the telephone screener survey will have to interview 770 problem gamblers. Data from one recent survey of the Ontario population (Wiebe, Mun, & Kaufman, 2006) has indicated that 5.5% of the adult population are problem gamblers (4.2% have moderate problems and 1.3% meet criteria for severe problems). In order to reduce the number of people needed to be screened in order to identify 770 problem gamblers, the telephone survey will only be conducted with adults who say they have spent more than $100 on gambling in the last year (25% of the general population). Thus, the screener will need to interview somewhere between 3,600 to 4,000 respondents to identify 770 problem gamblers (770/.055 = 14,000; assuming all problem gamblers spent more than $100 in the last year, 14,000 X .25 = 3,600; allowing an additional 400 interviews as a safety cushion for a total of 4,000 interviews).

Also important is to ensure that there are enough females in the sample to conduct the proposed sex difference analyses. Approximately a third of problem gamblers in the general population are female (Wiebe, Mun, & Kaufman, 2006). With the assumption that this proportion would be maintained in our sample (i.e., those who would be interested in self-help materials and agree to participate in the study), roughly 48 of the 144 respondents would be female. A sample with 24 respondents per condition is sufficiently large to conduct the proposed analyses. However, our experience using these same recruitment methods in other studies is that females tend to be more interested in self-help materials than males and also, to be willing to participate in the proposed research. Thus, we fully anticipate that more than a third of respondents will be female (perhaps as many as half will be female if the recruitment trends in our other studies hold true with this trial as well).

*Why choose a population survey as a recruitment strategy?*

There are a number of advantages and one disadvantage to choosing a population telephone survey as a recruitment strategy for evaluating a brief intervention. The disadvantage is that it is fairly expensive. However, we believe that the benefits outweigh the costs. In fact, the cost is not that much greater than a more traditional, newspaper recruitment method because newspaper recruitment requires more research staff to run. For newspaper recruitment, there must be a research assistant (RA) available to take calls from interested people and to screen for eligibility. Such an RA should be available during standard business hours at a minimum but, more ideally, in a big study such as the proposed one, an extra staff member should be available to take calls during the evening. These staffing costs can get expensive and even approach the costs of a population survey method if recruitment continues for an extended period. We believe the recruitment period would continue for an extended period because recruiting for evaluations of self-help interventions in Ontario is extremely difficult. The reason for this difficulty is unclear but it may be because research trials of this type conducted in other provinces (such as Alberta) mostly recruit problem gamblers who have trouble with VLTs (which are illegal in Ontario). As a concrete example, Dr. Toneatto attempted to recruit participants for a trial to evaluate the efficacy of a self-help book. He tried many different advertisements in different newspapers and even hired an advertising agency to develop advertisements that were ‘guaranteed’ to work because of their professional appearance. After spending $30,000 to recruit 12 participants, he had to conclude that the study could not be run. The alternate method of recruiting people who have already agreed to take part in another study, such as the use of a population survey, has had success in overcoming this reluctance of participating in a ‘treatment study.’ Participants in the population survey will have told us that they are interested in the personalized feedback materials. This question has been evaluated already (in our ongoing barriers to treatment study) and respondents are happy to answer the question and treat it as hypothetical (i.e., they are not expecting anything). We then go ahead and ask people who are hypothetically interested in these self-help materials if they would be interested in helping us develop and evaluate self-help materials for gamblers. This is an excellent way of getting problem gamblers to look at and try out materials without labelling the process as a treatment or intervention study. Thus, it gets around the barrier of trying to recruit people specifically for a treatment study. Another advantage of this recruitment script is that respondents who get assigned to the control condition are not actually surprised when they do not receive intervention materials. This lack of surprise is important and underlines the limitation of a standard waiting list control design; mainly, that if you recruit people for an intervention trial (self-help or otherwise) then they are expecting to receive an intervention. This is a major strength of a two-stage recruitment method, such as using a population survey, and one that cannot be achieved using a standard newspaper recruitment (i.e., you can’t recruit people who you know are interested in self-help materials who would be willing to participate in a trial without having them expect that they will get intervention materials – at least I can’t think of a way to do this). A major advantage of a population survey is that it allows for participants from all areas of Ontario, increasing the validity of the findings. This is an important issue, particularly as we have such promising results already (making it reasonable to move towards a design that has more of an effectiveness element to it). Also, the data collected as part of a population survey can have a number of other research purposes in addition to acting as a recruitment screener (i.e., produces data that is publishable in its own right – we outline some of these planned publications in the dissemination section). Finally, we have demonstrated a high level of interest in personalized feedback materials for problem gamblers using similar general population telephone screening methods (37.5% of problem gamblers said they would be interested). As such, we are confident in the utility of this telephone recruitment procedure.

**References**
